# Development and validation of a novel PCR-RFLP based method for the detection of 3 primary mitochondrial mutations in Leber's hereditary optic neuropathy patients

**DOI:** 10.1186/s40662-015-0028-0

**Published:** 2015-10-25

**Authors:** Siobhan Eustace Ryan, Fergus Ryan, David Barton, Veronica O’Dwyer, Derek Neylan

**Affiliations:** National Optometry Centre, Dublin Institute of Technology, Kevin Street, Dublin 8, Ireland; School of Biological Sciences, Dublin Institute of Technology, Kevin Street, Dublin 8, Ireland; Centre for Medical Genetics, Our Lady’s Hospital for Sick Children, Crumlin, Dublin 12, Ireland

**Keywords:** LHON, Mitochondrial mutations, Mutation detection, Visual morbidity, Multiplex PCR

## Abstract

**Background:**

Leber’s Hereditary Optic Neuropathy (LHON; MIM 535000) is one of the most commonly inherited optic neuropathies and it results in significant visual morbidity among young adults with a peak age of onset between the ages of 15–30. The worldwide incidence of LHON is approximately 1 in 31,000. 95 % of LHON patients will have one of 3 primary mitochondrial mutations, G3460A (A52T of ND1), G11778A (R340H of ND4) and T14484C (M64V of ND6). There is incomplete penetrance and a marked gender bias in the development of visual morbidity with approximately 50 % of male carriers and 10 % of female carriers developing optic neuropathy. Visual recovery can occur but is dependent on the mutation present with the highest level of visual recovery seen in patients who have the T14484C mutation. The 3 primary mutations are typically identified by individual end-point PCR-restriction fragment length polymorphism (RFLP) or individual targeted bi-directional Sanger sequencing reactions. The purpose of this study was to design a simple multiplex PCR-RFLP that could detect these 3 primary LHON mutations in one assay.

**Methods:**

PCR primers were designed to incorporate a MaeIII restriction site in the presence of 3460A and 14484C mutations with the 11778A mutation naturally incorporating a MaeIII site. A multiplex PCR-RFLP assay was developed to detect the 3 common mutations in a single assay. Synthetic LHON controls based on the mitochondrial genome harbouring the 3 common mutations were synthesized and cloned into plasmids to act as reliable assay controls. DNA from previously tested patients and the synthetic LHON controls were subjected to the multiplex PCR-RFLP assay. The RFLP products were detected by agarose gel electrophoresis.

**Results:**

The novel PCR-RFLP assay accurately detects the 3 primary mutations both in patient DNA and in synthesized DNA control samples with a simple visual mutation detection procedure. The synthesized DNA was demonstrated to be a robust control for the detection of LHON Mutations.

**Conclusion:**

In this paper, we describe a novel, robust and simple PCR-RFLP based method for the detection of mutations causing LHON, and report the generation of a series of LHON DNA controls suitable for all currently published assays.

**Electronic supplementary material:**

The online version of this article (doi:10.1186/s40662-015-0028-0) contains supplementary material, which is available to authorized users.

## Background

Leber’s hereditary optic neuropathy (LHON; MIM 535000) is one of the most commonly inherited optic neuropathies and it results in significant visual morbidity among young adults [1, 2]. The disorder is the result of mitochondrial dysfunction wherein primary mitochondrial DNA (mtDNA) mutations affect complex I subunits of the respiratory chain [3]. LHON is the most common among primary mitochondrial diseases with a prevalence of 1 in 31,000 in the North of England, 1 in 39,000 in the Netherlands and 1 in 50,000 in Finland [4–6]. In some countries, approximately 95 % of individuals with LHON have one of 3 primary mtDNA mutations; G3460A (A52T of ND1), G11778A (R340H of ND4) and T14484C (M64V of ND6) [3, 7, 8] while other rare mutations (such as G13730A, G14459A, C14482G, A14495G, C14498T, C14568Tand T14596A) account for the final 5 % [9–16]. LHON demonstrates marked gender bias and an incomplete penetrance, with approximately 50 % of males and 10 % of females who harbour one of the above mutations actually developing optic neuropathy [17–19]. This indicates that environmental or other genetic factors must play a role in penetrance. Alcohol consumption, smoking, certain prescription medications, stress and critical illness have been implicated in the onset of symptoms in LHON mutation carriers [17, 18, 20, 21].

The peak age of LHON onset is between 15–30 years and 95 % of carriers who will experience visual failure will do so before the age of 50 years [22]. However, visual deterioration can occur any time during the first to the seventh decade of life [23]. Clinically, there tends to be an acute loss of vision in one eye generally followed by loss of vision in the other eye within 8 weeks. In the majority of cases, LHON pathology is limited to a highly specialized group of cells within the eye known as retinal ganglion cells (RGCs). Histological analysis of the optic nerve in LHON patients reveals minimal evidence of any inflammation, but shows general axonal depletion centrally and fibrocytic scarring. Any residual axons were limited to superior and temporal peripheral clusters [24].

Visual recovery can occur in some LHON patients, but the extent of which is influenced by the kind of mutation involved in the development of a particular patient’s LHON. The highest level of visual recovery is seen with patients who have the T14484C mutation (up to 58 %), followed by those with the G3460A mutation (up to 25 %). Patients who harbour the G11778A mutation have the lowest level of visual recovery [25–28]. Thus, an accurate mutation detection strategy can have a significant prognostic value to the LHON patient.

Current diagnostic strategies for the 3 most common mutations causing LHON include individual endpoint PCR-RFLP [29], allele specific PCR [30], real time PCR [31] and PCR followed by Sanger / pyrosequencing [32]. In this study, we successfully designed a simple multiplex PCR-RFLP to detect the 3 primary mitochondrial LHON mutations and also describe the synthesis of a series of LHON control sequences that act as a robust and patient-free resource for LHON test controls and assay development.

## Methods

### Patient DNA

Patient DNAs were obtained from the Centre for Medical Genetics, Our Lady’s Hospital for Sick Children, Dublin, Ireland and Oxford Medical Genetics Laboratories, Oxford, UK. DNA was extracted from peripheral blood using the Centra Puregene Blood Kit (Qiagen, Manchester, UK) or the EZ1 Blood Kit on the EZ1 advanced XL instrument (Qiagen, Manchester, UK) according to the manufacturers’ instructions. All samples used in this study were previously tested for LHON mutations using PCR amplification and DNA sequencing. To maintain patient confidentiality during this study, aliquots of residual DNA material from the diagnostic test were labelled with the LHON mutation detected and irreversibly anonymised. The use of patient DNA in this study has received ethical approval from the Dublin Institute of Technology Research Ethics Committee (RN: 14–06).

### Synthetic control DNA

To provide an unlimited, reliable and patient-free resource for LHON testing across all current testing platforms as well as to allow for the development of the multiplex PCR-restriction fragment length polymorphism (RFLP) test described in this study, LHON control sequences were synthesised and cloned into standard plasmids by Eurofins Genomics (London, UK) or Life Technologies (Carlsbad, USA) based on the reference sequence NC_012920.1. A total of 6 plasmids were generated containing the 3460G, 3460A, 11778G, 11778A, 14484 T and 14484C sequences. The 3460 plasmids contained mitochondrial sequences from 3275 to 4272, the 11778 plasmids contained mitochondrial sequences from 11580 to 12118 and the 14484 plasmids contained mitochondrial sequences from 14449 to 15022 (Table 1). For the generation of synthetic diagnostic controls, the plasmids were combined at a concentration of 1.5 ng/μl to generate mixes containing none or one of the primary LHON mutations as follows; Normal control that contained no LHON mutations (a mix of 3460G, 11778G and 14484 T plasmids at a concentration of 1.5 ng/μl), 3460A control that contained 3460A, 11778G and 14484 T plasmids, 11778A control that contained 3460G, 11778A, 14484 T plasmids, and 14484C control that contained 3460G, 11778G, 14484C plasmids. This information is summarised in Table 1.

**Table 1:** Synthetic LHON control plasmids and mixes

Plasmid	Nucleotide Positions	Normal Mix	3460A mix	11778A mix	14484C mix
3460G (N)	3275 – 4272	X		X	X
3460A (M)	3275 – 4272		X		
11778G (N)	11580 – 12118	X	X		X
11778A (M)	11580 – 12118			X	
14484 T (N)	14449 – 15022	X	X	X	
14484C (M)	14449 – 15022				X

The table presents nucleotide positions, based on the reference sequence NC_012920.1, included in the cloned mitochondrial plasmids. 3460G indicates the normal G nucleotide at position 3460 and 3460A indicates the mutated A at position 3460; similarly, for 11778 and 14484 plasmids. The synthetic diagnostic control mixes are generated by mixing the indicated plasmids at 1.5 ng/μl. For a diagnostic test, 1 μl of a diagnostic control mix is used

### Primer design and PCR

Primers (Sigma Genosys, Arklow, Ireland) for the multiplex PCR-RFLP (Table 2) were designed to incorporate a MaeIII (Roche, Burgess Hill, UK, Catalogue Number 10822230001) restriction site (↓GTnAC) in the presence of 3460A and 14484C mutations. MaeIII was chosen because the 11778 mutation naturally introduces a MaeIII restriction site and minor alterations of the PCR primers as shown in Fig. 2a and Table 2, the 3460A and 14484C mutations also introduce a MaeIII site to allow the development of a multiplex PCR and RFLP strategy based on the modified primers and MaeIII restriction enzyme. The sequences of the PCR products highlighting the positions of the oligonucleotice primers and the MaeIII restriction sites are shown in the Additional files  1, 2 and 3. In Fig. 2a, alterations in the primer sequences leading to the generation of a MaeIII site are shown in lower case. Additionally, the 3460 PCR product contains a naturally occurring MaeIII site at position 3736 to act as an internal control of complete restriction in the assay. This multiplex assay uses one PCR reaction and one restriction enzyme thus allowing for a simple and cost effective assay for the 3 common LHON associated mutations. The primers produce products as follows: 3460 F/R (333 bp), 11778 F/R (164 bp) and 14484 F/R (236 bp), respectively. In the presence of the mutated allele, the products change as follows: the 3460A mutation resulting in 279 bp, 28 bp and 26 bp (control of restriction), 11778A mutation producing 135 bp, 29 bp, and 14484C mutation producing 205 bp, 31 bp, respectively. PCR was performed in a reaction containing 50 ng of genomic DNA (or 1 μl of the synthetic control described above) using 1 unit of Platinum Taq polymerase (Life Technologies, Carlsbad, USA, Catalogue Number 10966–034), 100 μM dNTP (Life Technologies, Carlsbad, USA, Catalogue Number 10297–018), a primer mix containing 250 ng of 3460 F/R, 60 ng of 11778 F/R and 40 ng of 14484 F/R and standard PCR buffer containing 3.5 mM MgCl_2_ (Life Technologies, Carlsbad, USA, Catalogue Number 10966–034). PCR conditions were 95 °C for 5 min followed by 35 cycles of 95 °C for 30 s, 59 °C for 30 s and 72 °C for 30 s followed by a final extension at 72 °C for 5 min. Restriction with MaeIII was performed using a reaction containing 12 μl of PCR product, 12.5 μl of 2X MaeIII buffer and 0.5 μl (1 unit) of MaeIII for a minimum of 2 h at 55 °C. Restriction products were detected by electrophoresis on a 2.5 % agarose gel (Life Technologies, Carlsbad, USA, Catalogue Number 16500–100) containing 0.5 μg/ml ethidium bromide (Sigma Genosys, Arklow, Ireland, Catalogue Number E7637).

**Table 2:** Primer sequences for multiplex PCR-RFLP

Mutation	Forward Primer	Reverse Primer	Product Size
G3460A	5'-CCCCTACGGGCTACTACAACCCTTCGCTGtC	5'-GATAGTAGAATGATGGCTAG	333 bp
G11778A	5'-AGCAAACTCAAACTACGAACG	5'-TTACTAGCACAGAGAGTTCTC	164 bp
T14484C	5'-AATAGCCATCGCTGTAGTATATCCAAAGACAgtCA	5'-GTGCGAGAATAATGATGTATGC	236 bp

Lowercase letters indicate alterations introduced to the forward primers, which when in combination with the mutation, generate a MaeIII site. PCR product sizes are indicated

## Results

**Fig. 1:**
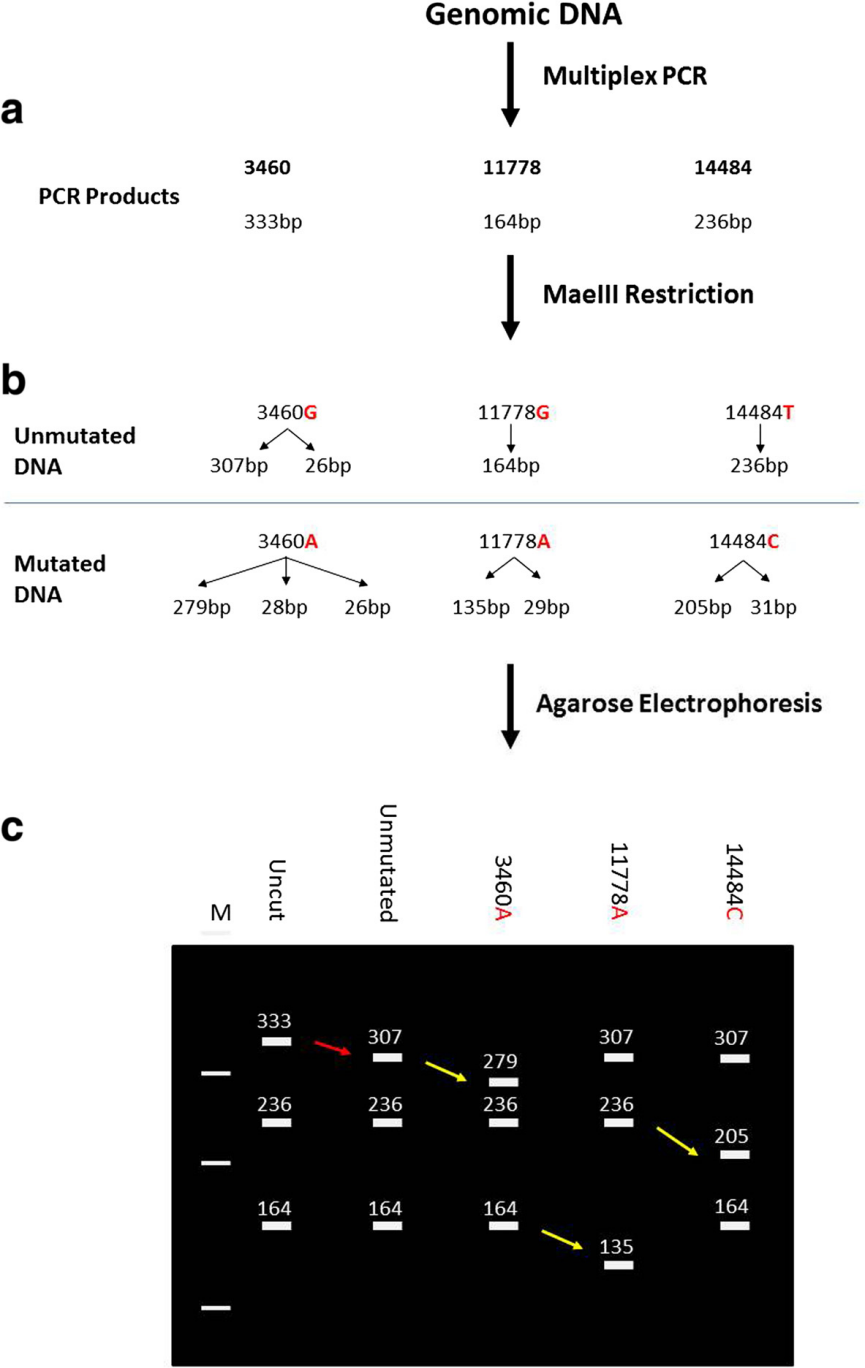
Workflow and expected results for the Multiplex PCR-RFLP. **a**: Sizes of PCR products generated in the multiplex PCR with products of 333 bp, 164 bp, and 236 bp. **b**: Size of restriction products generated by MaeIII restriction of the multiplex PCR products. In non-mutated samples, only the internal control of restriction in the 3460 product is restricted resulting in the removal of 26 bp from the 333 bp PCR product. **c**: Schematic representation of the band pattern expected from the diagnostic test. DNA samples with the 3460A, 11778A and 14484C mutations restricted with MaeIII yield the restriction products indicated. The red arrow indicates the control of restriction and the yellow arrows indicate mutation detection

The workflow and expected results are presented schematically in Fig. 1. As seen in Fig. 1, three PCR products are generated in the multiplex reaction with sizes of 333 bp, 164 bp and 236 bp for the 3460, 11778 and 14484 products, respectively. Figure 1b demonstrates the sizes of the restriction products generated from non-mutated samples and the sizes of restriction products generated from 3460, 11778 and 14484 mutated samples. Figure 1c depicts schematically the expected results from a diagnostic test on an agarose gel.

**Fig. 2:**
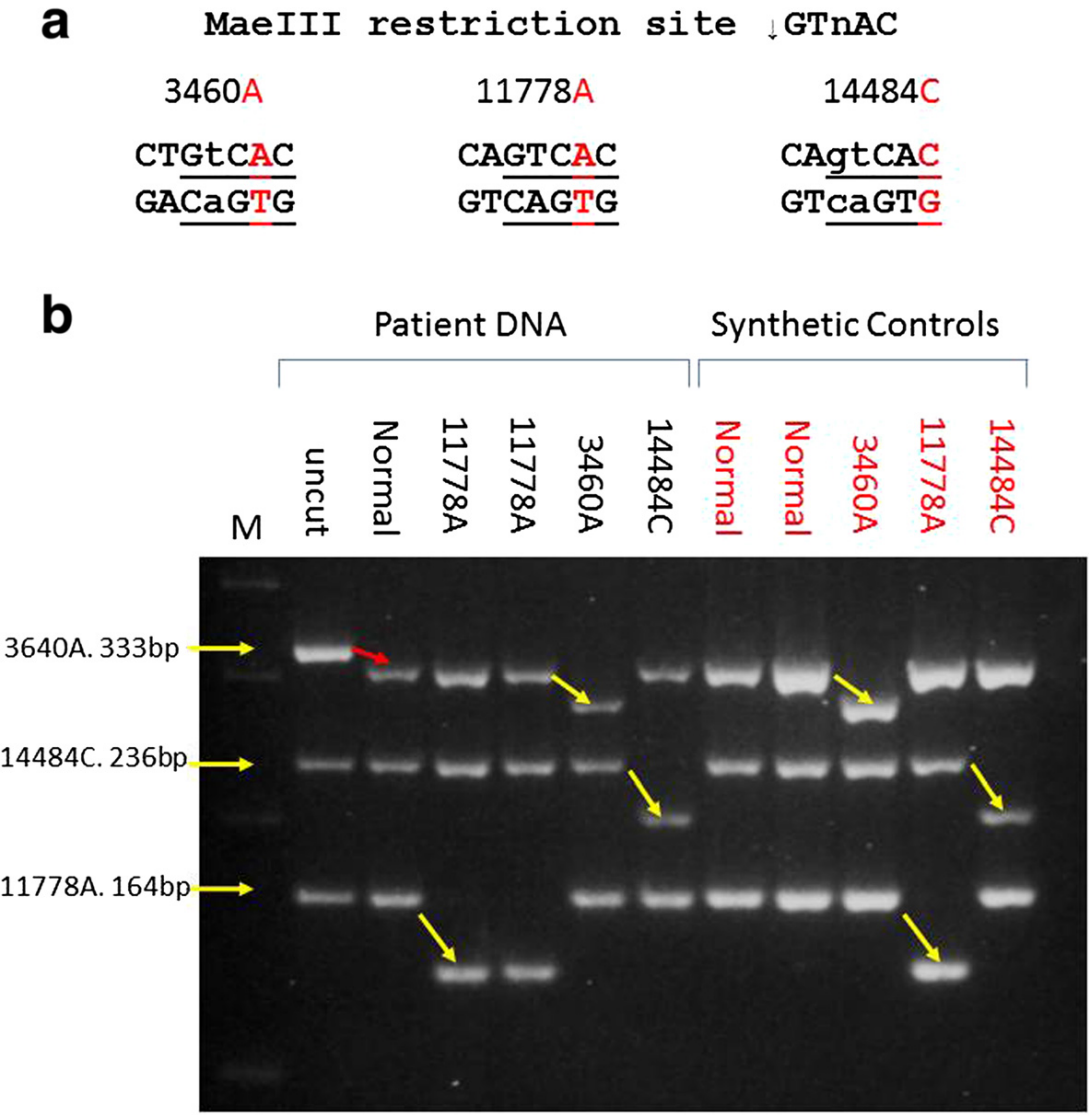
Multiplex PCR-RFLP. **a**: Diagram demonstrating the introduction of MaeIII restriction sites by the mutation (11778A) and the combination of primer alterations and mutation (3460A, 14484C). Mutations are shown in red with the primer alterations in lower case. **b**: 2.5 % ethidium bromide stained agarose gel showing the results of the PCR-RFLP on patient DNA (black labels) and synthesised DNA controls (red labels). DNA was PCR amplified in a multiplex reaction using 3460 F/R, 11778 F/R and 14484 F/R and restricted using 1 unit of MaeIII as described. M: size marker; Uncut: non-restricted PCR products; All other lanes: patient (Black) or synthesised DNA controls (Red) containing the indicated mutation PCR amplified and restricted with MaeIII. The red arrow between the uncut and normal lanes demonstrates the internal control of restriction. Yellow arrows demonstrate mutation detection

A range of patient DNA samples, non-mutated patient DNA samples, and synthetic control samples were tested in one assay using the multiplex PCR-RFLP as described above. The sizes of restriction products expected are indicated in Table 3 and Fig. 1. The results of this analysis are presented in Fig. 2b. All patient DNA and synthetic controls were genotyped by a blinded researcher and were correctly genotyped using the assay. The naturally occurring MaeIII site in the 3460 PCR product is used as an internal control of restriction and removes 26 bp from the PCR product. This can be seen between the uncut PCR product and the normal sample (lanes 2 and 3) and demonstrates a complete restriction in all lanes. The removal of the 26 bp from the 3460 PCR product confirms complete restriction by MaeIII and assures accurate mutation detection. The mutations are detected by the MaeIII restriction with the removal of 28 bp, 29 bp and 31 bp from the 3460, 11778 and 14484 PCR products, respectively. In all cases, the identification of the presence of a mutation is easily visualised on the 2.5 % agarose gel (Fig. 2b). The patient controls and synthetic control samples behave identically indicating that the synthesized DNA controls are a viable alternative to patient DNA for controls.

**Table 3:** PCR product and restriction product sizes expected in diagnostic test

1	2	3	4	5
Uncut PCR products	MaeIII Non-mutated	MaeIII 3460A	MaeIII 11778A	MaeIII 14484C
333	307	279	307	307
236	236	236	236	205
164	164	164	135	164
		28	29	31
	26	26	26	26

Column 1: PCR product sizes (bp) generated in the multiplex PCR. Column 2: Size of MaeIII restriction products obtained from non-mutated DNA samples. Column 3: Size of MaeIII restriction products obtained from 3460A samples. Column 4: Size of MaeIII restriction products obtained from 11778A samples. Column 5: Size of MaeIII restriction products obtained from 14484C samples

**Fig. 3:**
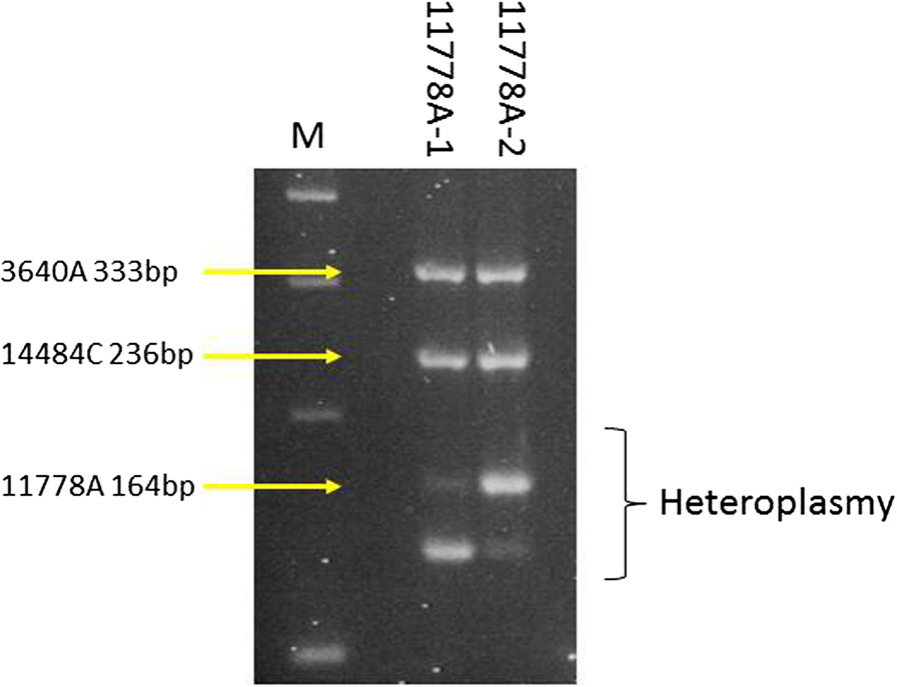
Detection of heteroplasmy using multiplex PCR-RFLP. 2.5 % ethidium bromide stained agarose gel showing the results of the multiplex PCR-RFLP on patient DNA heteroplasmic for G11778A mutation. M: size marker; 11778A-1: patient sample approximately 90 % 11778A; 11778A-2: patient sample approximately 10 % 11778A

As heteroplasmy is common with mitochondrial mutations, a mutation detection strategy must be capable of detecting heteroplasmy. Figure 3 presents an assay on heteroplasmic 11778 samples with approximately 90 % 11778A and approximately 90 % 11778G, indicating that the assay can accurately detect the presence of heteroplasmy to a level of approximately 10 %. Complete restriction of the naturally occurring MaeIII site in the 3460 PCR product again assures that the restriction is complete and that heteroplasmy was accurately detected.

## Discussion

This study aimed to develop a novel PCR-RFLP based multiplex assay for the detection of the 3 common primary mutations leading to Leber hereditary optic neuropathy (LHON). Approximately 95 % of LHON patients will have one of these 3 mutations, G3460A (13 %), G11778A (69 %) and T14484C (14 %). In approximately 110 diagnostic tests conducted in Ireland, only the 11778A mutation has been detected (data not shown). The PCR-RFLP approach was chosen because it provides a simple, cost effective, robust and easy to read output with minimal requirement for advanced technology. It also allows the detection of all three primary mutations in one multiplex analysis and allows the detection of heteroplasmy to a level of approximately 10 %. This is a significant advantage over individual endpoint PCR-RFLP [29] that requires 3 simplex PCRs followed by digestion with 3 separate restriction enzymes and electrophoresis. Allele-specific PCR [30], either simplex that requires 3 separate PCRs, or multiplex that requires only one PCR are efficient, but will not detect heteroplasmy in currently published formats. Individual real-time PCRs have been reported [31], but require more advanced technologies and are more costly. Individual endpoint PCR followed by bidirectional Sanger/pyrosequencing require significantly more hands-on time, and despite the decreasing costs of sequencing, are still significantly more expensive than the test reported here. As can been seen in Figs. 2b and 3, mutation identification is clear and robust, both with patient DNA and with the synthesised controls described above.

The genetic testing registry (http://www.ncbi.nlm.nih.gov/gtr/) (accessed July 2015) shows that the vast majority of LHON testing involves uni- or bi-directional Sanger sequencing targeted to the 3 primary mutations (*n* = 20), targeted simplex PCR-RFLP (*n* = 8), and other testing methods (including real-time mutation detection, PCR followed by hybridisation and pyrosequencing) (*n* = 4) with 2 laboratories offering a targeted 37 mitochondrial gene Next generation sequencing (NGS) panel (mtSEEK®). PCR followed by Sanger/Pyrosequencing can be used for LHON mutation detection, but requires individual PCRs followed by individual sequencing reactions, and despite decreasing costs for sequencing, still costs significantly more than the test described in this study. NGS with appropriate panels, will detect the 3 primary mutations [33] but requires significantly more hands-on time for both set up and bioinformatics analysis and, at present, is unlikely to be used for the detection of known mutations due to the costs involved and the simplicity of alternative tests. NGS would, however, be invaluable in the detection of rarer / unknown mutations post initial screening for the 3 primary mutations and could be used for suspected LHON patients negative for the primary mutation screen. The assay reported in this study will allow diagnostic laboratories to avoid costly NGS assays for the vast majority of LHON patients and allow resources to be focussed on patients with unknown mutations requiring further analysis. We suggest that the test described in this study will allow detection of the 3 primary LHON mutations in a single test format at minimal cost, with a rapid turnaround time and without the need for advanced technology.

It is currently estimated that 35,000 individuals worldwide are vision-impaired due to LHON. With the addition of extended family members to this figure, there is a significant requirement for a simple cost-effective and robust diagnostic strategy for LHON mutation detection. The steps required to obtain approval for this diagnostic test for clinical applications include validation of the strategy in larger scale trials in multiple laboratories to ensure reproducibility and sensitivity/specificity followed by applications to the national and international competent authorities such as FDA and EU medical devices sections.

We also describe the generation of a series of controls for LHON applicable for all currently described LHON testing algorithms and demonstrate their applicability in this novel test. The generated controls have also been tested in simplex PCR-RFLP (as described in Marotta et al. 2004) and ARMS PCR (as described in Bi et al. 2010) – data not shown. The controls will provide an unlimited, reliable, and patient-free resource for LHON testing across all current testing platforms. This resource may allow the development of further tests in the future.

## Conclusion

We developed a novel, cost-effective multiplex PCR-RFLP based assay for the detection of the 3 most common mutations causing LHON and demonstrated the robustness of the assay in patient and synthetic controls. The assay provides a significant advantage over simplex PCR-RFLP and Sanger/pyrosequencing approaches to mutation detection in terms of costs and hands-on time required. A series of cloned LHON and normal controls were developed and their uses in this and other testing strategies confirmed. This will be a useful resource for future test development and diagnostic laboratories as it provides an unlimited and patient-free source of control material.

## Additional files

Additional file 1:
**11778 sequence.** (JPEG 68 kb) Additional file 2:
**14484 sequence.** (JPEG 74 kb) Additional file 3:
**3460 sequence.** (JPEG 82 kb)
