# Supplementary figures and images for: Development and validation of a novel PCR-RFLP based method for the detection of 3 primary mitochondrial mutations in Leber's hereditary optic neuropathy patients
Source: Eye Vis (Lond). 2015 Oct 25;2:18. doi: 10.1186/s40662-015-0028-0 (PMC4657363; doi:10.1186/s40662-015-0028-0)

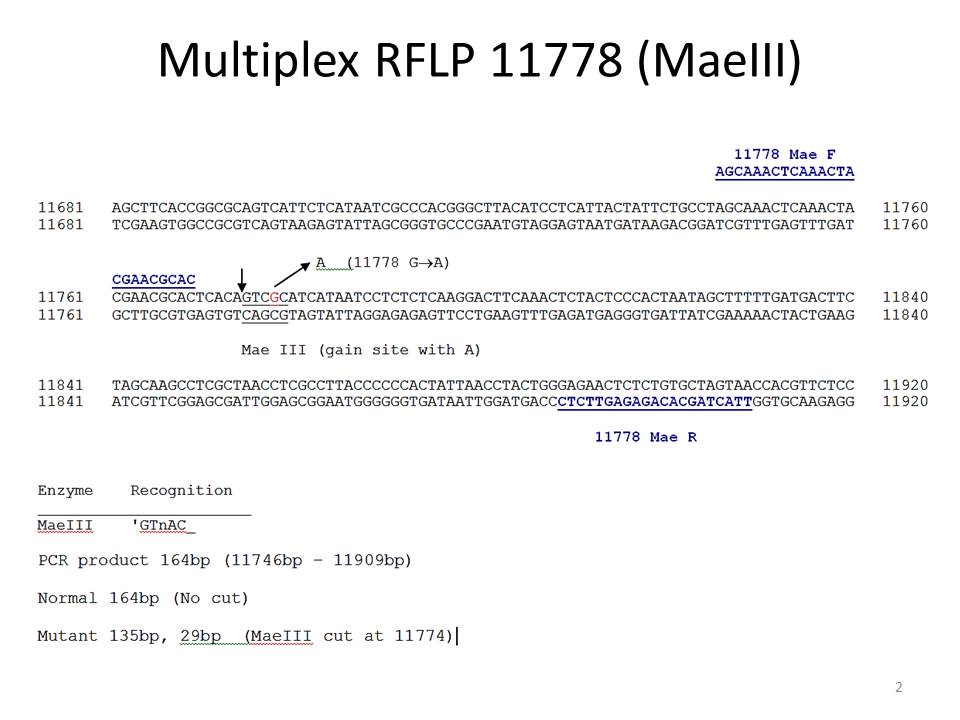

Supplement: Additional file 1: — 11778 sequence. (JPEG 68 kb) [file 40662_2015_28_MOESM1_ESM.jpg]

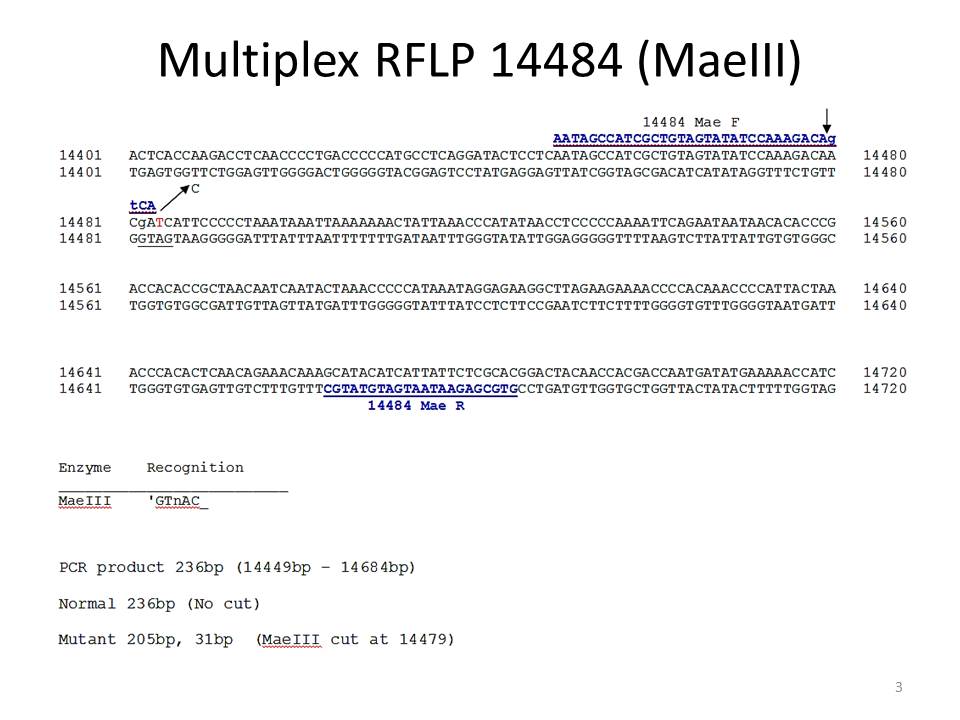

Supplement: Additional file 2: — 14484 sequence. (JPEG 74 kb) [file 40662_2015_28_MOESM2_ESM.jpg]

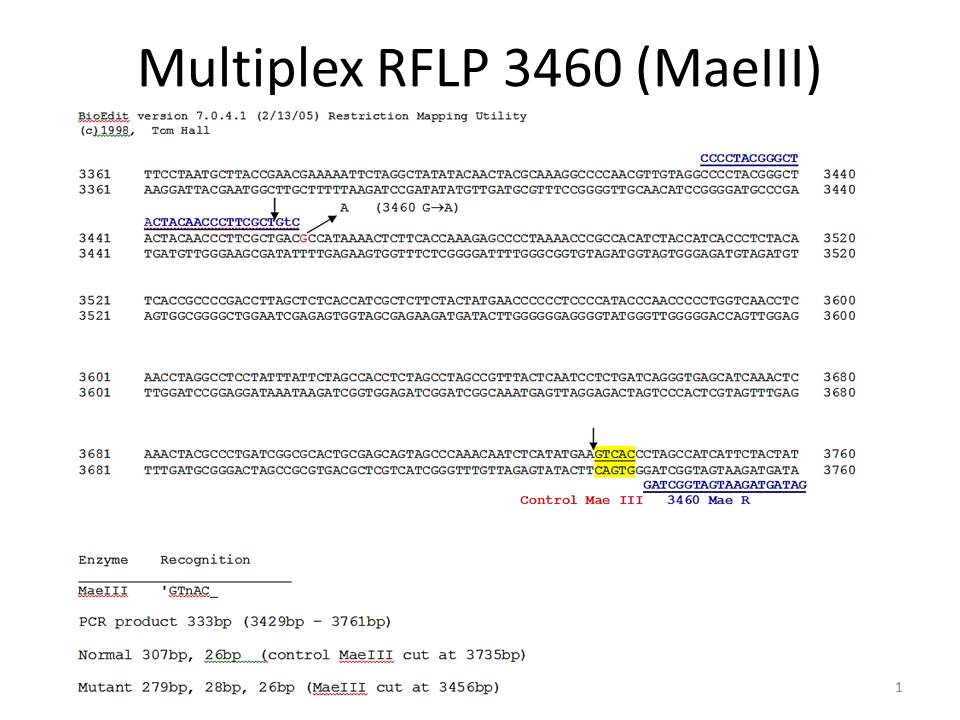

Supplement: Additional file 3: — 3460 sequence. (JPEG 82 kb) [file 40662_2015_28_MOESM3_ESM.jpg]
